# Depression in quarantined patients during the COVID-19 pandemic

**DOI:** 10.1192/j.eurpsy.2021.753

**Published:** 2021-08-13

**Authors:** A. Guermazi, J. Ben Thabet, A. Zouari, J. Aloulou, R. Hammami, H. Ben Ayed, A. Sallemi, C. Marrekchi, S. Hdiji, I. Gargouri, M. Kassis, M. Turki, S. Kammoun, M. Larbi Masmoudi

**Affiliations:** 1 Psychiatry C Department, Hedi chaker University hospital, sfax, Tunisia; 2 Department Of Paychiatry C, Hedi chaker hospital, Sfax, Tunisia; 3 Psychiatry (b), Hedi Chaker University hospital, sfax, Tunisia; 4 Cardiology Department, Hedi chaker University hospital, sfax, Tunisia; 5 Community Health And Epidemiology Department, Hedi chaker University hospital, sfax, Tunisia; 6 Histology Department, Hedi chaker University hospital, sfax, Tunisia; 7 Infectiology Department, Hedi chaker University hospital, sfax, Tunisia; 8 Hematology Department, Hedi chaker University hospital, sfax, Tunisia; 9 Occuaptional Department, HediChaker Hospital, sfax, Tunisia; 10 Community Health And Epidemiology Department, HediChaker Hospital, sfax, Tunisia; 11 Pneumology, HediChaker Hospital, sfax, Tunisia; 12 Department Of Occupational Medicine, HEDI CHAKER hospital, SFAX, Tunisia

**Keywords:** quarantine, suspected patients, Depression, COVID-19 pandemic

## Abstract

**Introduction:**

Quarantine for suspected patients of being infected by the COVID-19 can lead to negative consequences for mental health and the appearance of depressive symptoms.

**Objectives:**

To assess the prevalence of depression in quarantined patients, and to analyze the associated factors.

**Methods:**

This was a descriptive and analytical survey, carried out from April 4 to May 30, 2020, with 149 patients consulting the COVID-19 sorting box at the Hedi Chaker CHU in Sfax. Suspected COVID-19 patients were contacted by phone during their quarantine and invited to participate in our study. The Patient Health Questionnaire (PHQ-9) scale was used to assess the severity of depression. Cutoffs of 5, 10, 15, and 20 represent minimal, mild, moderate, moderately severe, and severe levels of depression based on PHQ-9 scores. A cutoff score of 10 determines major depression.

**Results:**

The results showed a prevalence of major depression of 10.7%. Of all patients, 89.3% had minimal to mild depression; 10% had moderate to moderately severe depression and 0.7% had severe depression. The PHQ-9 score was statistically correlated with travel to a suspect area during the 14 days preceding the consultation (p = 0.008), contact with a subject confirmed COVID-19 (p = 0.01), previous follow-up in psychiatry (p = 0.047), the change of residence during quarantine (p = 0.045), the fear of transmitting the disease to relatives (p = 0.00) and the positive result of the nasopharyngeal swab (p = 0.00).

**Conclusions:**

Psychological distress was felt in our patients. We recommend that necessary measures should be taken to combat depression.

